# A Novel Open-Framework Cu-Ge-Based Chalcogenide Anode Material for Sodium-Ion Battery

**DOI:** 10.1155/2017/3876525

**Published:** 2017-12-27

**Authors:** Quan Sun, Lin Fu, Chaoqun Shang

**Affiliations:** ^1^School of Materials Science and Engineering, Tongji University, Shanghai 201804, China; ^2^Key Laboratory of Electrochemical Energy Storage Technology of Jiangsu Province, Taizhou 225500, China; ^3^Department of Materials Science and Engineering, Southern University of Science and Technology, Shenzhen 518055, China

## Abstract

Open-framework chalcogenides are potential electrode materials for sodium-ion batteries (SIBs) due to their architectures with fast-ion conductivity. Herein, we report on the successful synthesis of open-framework Cu-Ge-based chalcogenides [Cu_8_Ge_6_Se_19_](C_5_H_12_N)_6_ (CGSe) and the research of their energy storage application as SIB anodes for the first time. As a result, the CGSe anode exhibited good electrochemical performances such as high reversible capacity (463.3 mAh g^−1^), excellent rate performance, and considerable cycling stability. Our exploration not only develops a promising electrode material for SIBs, but also extends the application of open-framework chalcogenides.

## 1. Introduction

Because of the identical fundamental principles, sodium-ion batteries (SIBs) are considered to be one of the most potential substitutes for lithium-ion batteries (LIBs) [1, 2]. Moreover, SIBs might become competitive with LIBs in large-scale storage applications owing to the abundance of sodium and shortage of lithium in the earth [3]. Unfortunately, compared with lithium ions (0.76 Å), the larger ionic radius of sodium ions (1.02 Å) is a big hurdle for intercalation reaction with anode materials [4]. For example, the commercial graphite delivers a sodiation capacity of less than 35 mAh g^−1^, which is several times lower than that of lithiation capacity (372 mAh g^−1^) [5]. It is worth celebrating that the anode materials with alloying reaction and conversion reaction mechanism exhibit high specific capacity for SIBs, such as Si, Ge, Sn, Sb, P and their compounds [6–9]. Ge has been found to alloy with Na at room temperature to form NaGe with a theoretical capacity of 369 mAh g^−1^ [10]. However, due to the sluggish bulk diffusion of Na ions, elemental Ge delivered high specific capacities only in thin film and amorphous structures, and the Na-ion storage properties of coarser structures remain limited [11–13]. Furthermore, the serious volumetric variation of Ge during the alloying and dealloying process will lead to fast decay of specific capacity. The preparation of compounds has been applied to overcome the above problems and improve the electrochemical performances of Ge-based anode materials. In recent years, the inorganic Ge-based compounds (such as GeO_2_/reduced graphene oxide (RGO), GeP_5_/C, Zn_2_GeO_4_/RGO, and ZnGeP_2_/C) have been proposed as anode materials for SIBs and have shown stable cycle property and high rate capability [14–17]. Nevertheless, the nanosize particles of these materials as well as the introduction of RGO or C in Ge-based compounds usually aggravate the side reaction with electrolyte and decrease the volume energy density of SIBs. Therefore, it is essential to search novel strategies to enhance the bulk Na ions diffusion and suppress the volume expansion during sodiation of Ge-based anode materials.

Open-framework chalcogenides have aroused intensive interest in visible-light photocatalysis over the past few decades because these materials can integrate porosity with semiconductivity [18]. More importantly, the open-framework chalcogenides with characteristics of three dimensional (3D) ion migration channels, high porosity, and high anionic framework polarizability have long been recognized as potential fast-ion conductors, which can be used as electrodes or electrolytes in secondary batteries [19]. The Ca-Li-In-S quaternary open-framework chalcogenides with the highest specific conductivity of 0.15 Ω^−1 ^cm^−1^ at 27°C under 100% relative humidity have been reported by Zheng et al. [20]. Recently, the crystalline chalcogenide (H_3_O)(enH_2_)Cu_8_Sn_3_S_12_ with frame structure has been investigated as anode material in lithium-ion batteries and exhibited a high initial reversible capacity of 870.3 mAh g^−1^ [21]. However, the electrochemical behavior of this family of materials for SIBs has not been reported. Lately, a Cu-Ge-S open-framework chalcogenide with 3D channels has been reported [22]. The cylindrical channel along the *c*-axis showed a diameter of 16.4 Å, which provided huge space for Na ions diffusion. Furthermore, the large Cu^+^ to Ge^4+^ ratio (Cu/Ge = 1.6) made this chalcogenide show low electronic band gap (2.5 eV). Moreover, the porous nature of open-framework chalcogenides would facilitate the penetration of electrolyte and transportation of ions and buffer the volume expansion during sodiation [23]. On the other hand, the conversion reaction and alloying reaction of Cu^+^ and Ge^4+^ with Na ions in this chalcogenide would exhibit high theoretical capacity. Therefore, the open-framework Cu-Ge-based chalcogenides are highly potential anode materials for SIBs.

In this work, we successfully synthesize a novel open-framework chalcogenide [Cu_8_Ge_6_Se_19_](C_5_H_12_N)_6_ (CGSe) and investigate its sodium-ion storage properties for the first time. The CGSe cubic crystals with 10–50 *μ*m edge length show good electrochemical performance, indicating that CGSe offers an opportunity to anode materials for high performance SIBs in the future.

## 2. Experimental Details

The CGSe samples were synthesized according to the literature methods [24]. In the typical synthesis process, 187 mg of Cu(Ac)_2_·H_2_O, 104 mg of GeO_2_, and 215 mg of Se were mixed in 2.5 mL of piperidine (17.3 wt%) solution under vigorous stirring for about 30 min. The reaction solution was then transferred to a 23 mL Teflon-lined stainless-steel autoclave and maintained at 180°C for 10 days. After cooling, the black cubic crystals were harvested by ultrasonic treatment, washed with ethanol, and dried at 70°C for 1 day.

The structure of as-prepared samples was determined by single-crystal X-ray diffraction (SCXRD, Agilent diffractometers) using graphite monochromated Mo K*α* radiation (*λ* = 0.71073 Å) with SHELXS-97 method. Its phase purity was supported by powder X-ray diffraction (PXRD, Bruker, D8 advance) using Cu K*α* radiation (*λ* = 1.5406 Å) with a step size of 0.3° in the 2 theta range 5–30°. The lattice structural details were acquired on a FEI Tecnai G^2^ F-20 high-resolution transmission electron microscope (HRTEM). The morphologies and corresponding elemental mapping images were obtained by field-emission scanning electron microscope (FE-SEM, Hitachi S-4800).

Na-ion storage properties of CGSe were evaluated using CR2032 coin-type half batteries. The working electrodes were prepared by coating a mixture containing CGSe (80 wt%), super P (10 wt%), and poly(acrylic) acid (PAA) binder (10 wt%) onto copper foil and dried at 120°C for 1 day. The coin-type cells assembled in recirculating argon glove box by using Na metal as counter electrode, glass microfiber filter as separator, and 1 M NaClO_4_ dissolved in ethylene carbonate (EC) and dimethyl carbonate (DMC) (1 : 1 by volume) with 5 wt% fluoroethylene carbonate (FEC) additive as electrolyte. Cyclic voltammetry (CV) data were collected on an electrochemical workstation (BioLogic VMP-300) at a scanning rate of 0.2 mV s^−1^ in the voltage range of 0.005–2.5 V versus Na^+^/Na. The cycle and rate properties tests were performed on a LAND battery measurement system (LAND CT2001A) between 0.005 V and 2.5 V. The mass loading of CGSe was about 1.5 mg cm^−2^ in the electrode and the specific capacities were calculated based on active materials.

## 3. Results and Discussion

**Figure 1:**
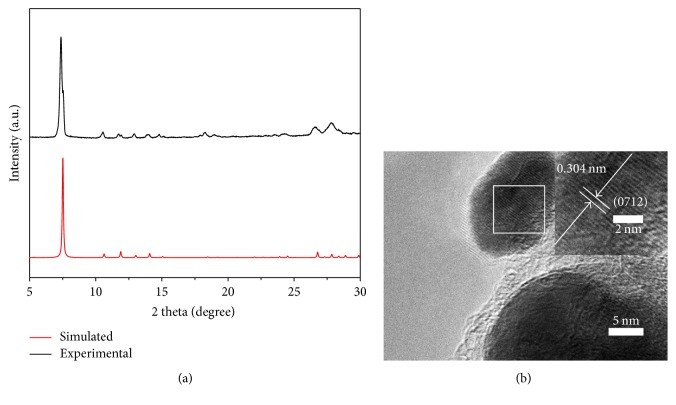
(a) XRD patterns and (b) HRTEM image of CGSe.

The CGSe crystallizes in the cubic space group* Im-3*. As depicted in Figure 1(a), all diffraction peaks of PXRD are well matched to the simulated ones from SCXRD analysis, indicating the highly pure single-crystal structure of as-prepared CGSe. In the HRTEM image (Figure 1(b)), the marked lattice distance of 0.304 nm can be assigned to the (0712) crystal plane of CGSe, which is in agreement with the XRD peak located at 28.8°. The HRTEM image further demonstrated that the crystal structure of as-prepared CGSe is well matched with the SCXRD result.

**Figure 2:**
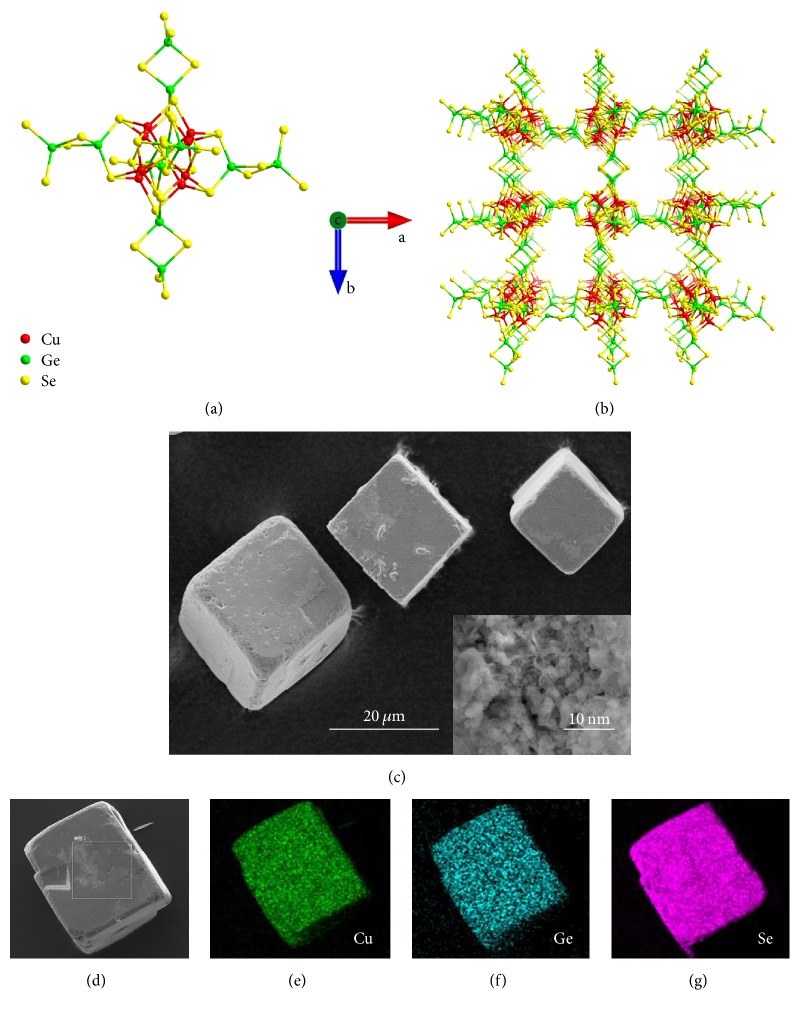
(a) The ball and stick mode of icosahedral [Cu_8_Se_19_]^24−^ cluster and its connectivity with twelve Ge^4+^ ions. (b) View of the CGSe framework along the ab direction. (c, d) SEM images and the corresponding EDS mapping of (e) Cu, (f) Ge, and (g) S of CGSe. The high-magnification SEM image inset in Figure 2(c).

In open-framework CGSe, the 3D interconnected channels are constructed with the anionic selenide framework [Cu_8_Ge_6_Se_19_]^6−^ and disordered charge-balanced species (C_5_H_12_N)^+^ [21, 24]. The inorganic species [Cu_8_Ge_6_Se_19_]^6−^ forms the porous structure, while the organic species (C_5_H_12_N)^+^ acts as the structure stabilizer. As displayed in Figure 2(a), the structural feature of 3D CGSe is the presence of icosahedral [Cu_8_Se_19_]^24−^ cluster, consisting of a cubic array of eight Cu^+^ ions bridged by Se^2−^ ions. The shape of [Cu_8_Se_19_]^24−^ cluster defined by nineteen Se^2−^ sites is icosahedral. To form the infinite lattice (Figure 2(b)), the primitive cubic packing of icosahedral [Cu_8_Se_19_]^24−^ clusters is cross-linked by dimeric Ge_2_Se_2_^4+^ unit. Most importantly, this extended CGSe open framework contains abundant interconnected microchannels, which is favorable to the sodium-ion intercalation. Furthermore, compared with Cu-Ge-S chalcogenide, the larger size of Se^2−^ compared to that of S^2−^ ions would make CGSe have much higher anionic framework polarizability, which is helpful to Na^+^ ions migration. The panoramic SEM image (Figure 2(c)) reveals that the CGSe samples are composed of cubic crystals, and the edge length of the single CGSe is about 10–50 *μ*m. It could be clearly seen from the high-magnification SEM image (inset in Figure 2(c)) that the inside of CGSe contains a great many of microchannels, which will facilitate the electrolyte infiltration and Na-ion diffusion in bulk CGSe. The energy dispersive X-ray spectroscopy (EDS) elemental mappings of Cu, Ge, and Se, as shown in Figures 2(e)–2(g), respectively, matched well with the corresponding SEM image (Figure 2(d)), indicating that these three elements are homogeneously distributed throughout the CGSe mass.

**Figure 3:**
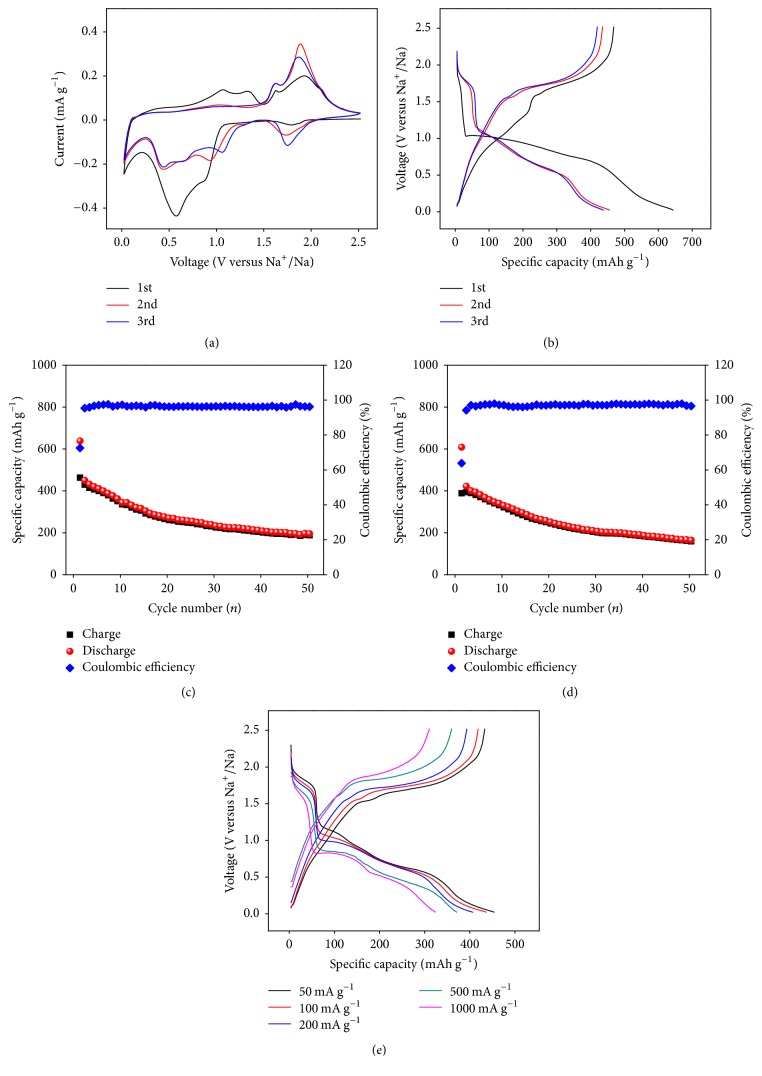
(a) The initial three curves of the CGSe at a scanning rate of 0.2 mV s^−1^. (b) The charge/discharge curves and (c) cycling performance of the CGSe at a current density of 100 mA g^−1^. (d) Cycling performance of the CGSe at a current density of 200 mA g^−1^. (e) The rate capability of CGSe under varying current densities (from 50 to 1000 mA g^−1^), respectively.

To explore the potential application of CGSe as SIB anode, we evaluated the electrochemical performances with the cyclic voltammetry (CV) and galvanostatic charge/discharge tests. The initial three consecutive CV curves of as-prepared CGSe are shown in Figure 3(a). In the reduction process, a cathodic peak centered at 1.7 V could be attributed to sodium-ion intercalation into the interconnected channels of CGSe and formation of Na_x_CGSe [25]. In the first discharge cycle, a weak peak at 0.85 V and a strong peak at 0.56 V were clearly observed, which can be attributed to the decomposition of Na_x_CGSe (Cu and Ge are generated) and formation of Na_y_Se, respectively [21, 26]. These peaks' shift to about 0.9 (1.1) and 0.4 V in the subsequent cycles is known to represent the activation of electrode materials during the first cycle [27]. Meanwhile, the intensities of peaks of the following two cycles are reduced, indicating the formation of solid electrolyte interface (SEI) film and other some irreversible reactions in the first cycle [28]. The cathodic/anodic couples at voltages of around 0.01/0.21 V might be assigned to signature of the NaGe alloying/dealloying reaction [16]. For the oxidation process, two smooth peaks were located at 1.05 V and 1.32 V in the first scan and disappeared in subsequent cycles because of the decomposition of instability SEI film. Two sharp peaks positioned at 1.61 V and 1.86 V can be associated with the desodiation process of the Na_y_Se and regeneration of CGSe, respectively [28–30].

The charge/discharge plateaus are in accordance with the CV results in Figure 3(b). The first charge/discharge curve is obviously different from the following two cycles. Moreover, the initial Coulombic efficiency (CE) is only 72.56% at a current density of 100 mA g^−1^. These abnormal electrochemical behaviors can be attributed to the SEI formation and other side reactions in the first cycle. In the following two cycles, the almost-overlapped charge/discharge curves as well as the high CE indicate the good reversible properties of CGSe, which should result from the unique morphology and structure of open-framework CGSe. The cycling performance of CGSe electrode was tested at 100 mA g^−1^ (Figure 3(c)). The charge capacity is 463.3 mAh g^−1^ in the first cycle and 188 mAh g^−1^ remains after 50 cycles. In addition, the CE increases to more than 96% (the third cycle) and then keeps stable during cycling. The initial reversible capacity is higher than that of theoretical value of elemental Ge, indicating that CGSe is a promising anode material for high energy density SIB. It is noteworthy that the CGSe anode materials can also exhibit reversible capacity of 159 mAh g^−1^ after 50 cycles at the current density of 200 mA g^−1^ (Figure 3(d)). The rate capability of CGSe is also investigated.  Figure 3(e) shows the third charge/discharge curves of CGSe at different current densities. The excellent rate property is demonstrated by the specific discharge/charge capacities being around 450/429, 432/414, 403/389, 366/355, and 320/306 mAh g^−1^ at current density of 50, 100, 200, 500, and 1000 mA g^−1^, respectively. More importantly, although polarization becomes more obvious at the higher current density, the symmetric charge/discharge plateaus are still clearly observed. According to the previous literature, the shorter edge length of 3D channel of open-framework CGSe is about 6.4 Å, which is several times larger than that of ionic radius of sodium ions (1.02 Å) [4, 24]. To the best of our knowledge, these large channels are beneficial for the penetration of electrolyte and insertion/extraction of sodium ion, thereby resulting in good electrochemical performance [21, 23].

## 4. Conclusions

In conclusion, the crystalline CGSe open-framework material was successfully prepared using a simple solvothermal method. The as-prepared microscale CGSe cubic crystals display high reversible capacity (463.3 mAh g^−1^), excellent rate performance, and considerable cycling stability as a novel anode for SIBs because the interconnected channels facilitate penetration of electrolyte and transportation of sodium ions. It is also noteworthy that if the cycling stability of CGSe was improved by further research, it will become a promising anode in SIB fields. This work not only develops a potential electrode material for SIBs, but also extends the application of open-framework chalcogenides. In the future, it is suggested to pay much attention to the Na-ion storage mechanism as well as the capacity fading mechanism and solutions of the open-framework chalcogenides.
